# Ulnar Wrist Pain Revisited: Ultrasound Diagnosis and Guided Injection for Triangular Fibrocartilage Complex Injuries

**DOI:** 10.3390/jcm8101540

**Published:** 2019-09-25

**Authors:** Wei-Ting Wu, Ke-Vin Chang, Kamal Mezian, Ondřej Naňka, Yi-Chiang Yang, Yu-Chun Hsu, Po-Cheng Hsu, Levent Özçakar

**Affiliations:** 1 Department of Physical Medicine and Rehabilitation, National Taiwan University Hospital, Bei-Hu Branch, Taipei 10845, Taiwanmyronrbman@gmail.com (P.-C.H.); 2 Department of Physical Medicine and Rehabilitation, National Taiwan University College of Medicine, Taipei 10845, Taiwan; 3 Department of Rehabilitation Medicine, Charles University, First Faculty of Medicine, 12800 Prague, Czech Republic; kamal.mezian@gmail.com; 4 Institute of Anatomy, Charles University, First Faculty of Medicine, 12800 Prague, Czech Republic; ondrej.nanka@lf1.cuni.cz; 5 Department of Physical Medicine and Rehabilitation, Taipei Veterans General Hospital, Taipei 11217, Taiwan; yichiang2312@gmail.com (Y.-C.Y.); viph062@gmail.com (Y.-C.H.); 6 Department of Physical and Rehabilitation Medicine, Hacettepe University Medical School, Ankara 06100, Turkey; lozcakar@yahoo.com

**Keywords:** triangular fibrocartilage complex, wrist, ultrasound, magnetic resonance imaging

## Abstract

The triangular fibrocartilage complex (TFCC) serves as the major stabilizer of the wrist. Its injuries can result from trauma or degeneration, both of which are strongly correlated with the loading stress on the ulnar shaft and carpal joints. The TFCC is made of the articular disc, meniscus homologue, ulnocarpal ligament, radioulnar ligament, ulnotriquetral ligament, ulnolunate ligament, and subsheath of the extensor carpi ulnaris tendon. Because of its complexity, it is challenging to confirm the exact component affected in TFCC injuries. The Palmer classification is widely used for investigation of TFCC lesions using magnetic resonance imaging. Recently, high-resolution ultrasound (US) has become more popular in diagnosing musculoskeletal disorders. However, the utility of US imaging in TFCC lesions is less common because its anatomy under US imaging is not described in the current literature. Accordingly, in this review, we aimed to propose a standard US scanning protocol for the TFCC, present relevant images for its pathologies, and illustrate appropriate US-guided injection techniques for their management.

## 1. Introduction

Triangular fibrocartilage complex (TFCC) injuries are the most common cause of ulnar wrist pain. The clinical findings include localized pain, swelling, limited range of forearm supination-pronation, and wrist instability. These injuries may be caused by ulnar impaction syndrome, radial or ulnar fractures, and ulnocarpal ligament injuries.

The TFCC serves as a major stabilizer of the distal radioulnar and ulnocarpal joints and provides cushion for the axial load of the forearm on the ulnocarpal joint [1]. It is formed by the articular disc, meniscus homologue, ulnocarpal ligament, dorsal and palmar radioulnar ligaments, ulnar collateral ligament, ulnotriquetral ligament, ulnolunate ligament, subsheath of the extensor carpi ulnaris (ECU) tendon [2], and prestyloid recess, making it a complex structure for evaluation [3].

To date, the diagnosis of TFCC lesions is still difficult even with the use of magnetic resonance imaging (MRI) [4]. Recently, high-resolution ultrasound (US) has emerged as a useful and valid tool for the diagnosis of musculoskeletal disorders [5,6,7]. Although several protocols have been published regarding US examination of the wrist [8] and nearby neural structures [9], there is limited information regarding how the TFCC can be systematically scanned. In this sense, this review aimed to propose a standard US scanning protocol for the TFCC, present exemplary images for its pathologies, and illustrate prompt US-guided injection techniques for their management.

## 2. Anatomy 

**Figure 1:**
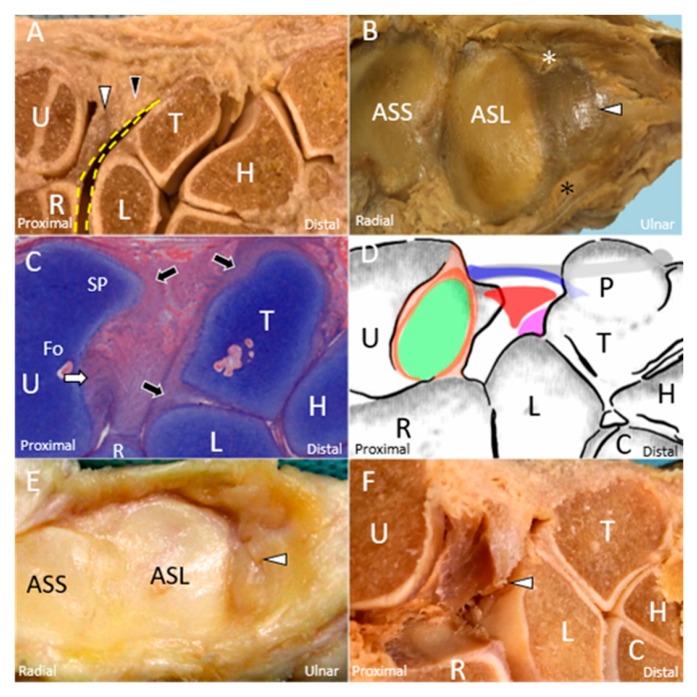
The cadaveric model shows the articular disc *(white arrowhead)* and meniscus homologue *(black arrowhead)*. (**A**) Between the disc and the carpal bones is the prestyloid recess *(yellow dashed line)*. (**B**) The disc *(white arrowhead)* is surrounded by the dorsal *(white asterisk)* and palmar *(black asterisk)* radioulnar ligaments. (**C**) The deep limb of the radioulnar ligament *(white arrow)* is inserted into the ulnar fovea. The cartilage *(black arrow)* of the ulna, lunate, and triquetrum can be clearly viewed. (**D**) The schematic drawing elaborates the triangular fibrocartilage complex, including the superficial limb of the radioulnar ligament *(light brown shade)*, deep limb of the radioulnar ligament *(dark brown shade)*, articular disc *(green shade)*, extensor carpi ulnaris tendon *(grey shade)*, ulnar collateral ligament *(blue shade)*, meniscus homologue *(red shade)*, and lunotriquetral ligament *(purple shade)*. The cadaveric model shows the (**E**) central tear and (**F**) radial avulsion of the articular disc *(white arrowhead)*. U: ulna; R: radius; L: lunate; T: triquetrum; P: pisiform; H: hamate; C: capitate; SP: styloid process; Fo: fovea; ASS: articular surface of the radius for the scaphoid; ASL: articular surface of the radius for the lunate.

The major component of the TFCC is the triangular-shaped articular disc. It is a fibrocartilage with a thicker peripheral region. The disc originates from the cartilage overlying the distal radius [10] and inserts onto the fovea of the ulnar styloid process. There are some fibrous tissues connecting the disc and the subsheath of the ECU tendon [11] (Figure 1A). The articular disc is surrounded by the dorsal and palmar radioulnar ligaments (Figure 1B), which are the primary stabilizers of the distal radioulnar joint (DRUJ). These ligaments have superficial and deep limbs, which are attached to the ulnar styloid process and its fovea, respectively (Figure 1C) [12,13]. The two limbs are separated by fibro-vascular tissues, i.e., ligamentum subcruentum [14]. The ulnar fovea is a bony depression between the hyaline cartilage of the distal ulna and the ulnar styloid [15]. Foveal attachment of the TFCC considerably contributes to the stability of the DRUJ and ulnocarpal joint [16].

Other structures within the complex include the meniscus homologue, ulnar collateral ligament, and ECU tendon subsheath, all of which are contributing to the stability of the TFCC (Figure 1D). Besides, at the palmar aspect of the wrist, the TFCC is reinforced by the ulnolunate and ulnotriquetral ligaments, both of which originate from the palmar radioulnar ligament [1,17].

## 3. Mechanism of Injury

The mechanism of TFCC injuries can be divided into traumatic and degenerative causes. Both strongly correlate with the load exerted on the ulnar shaft and carpal joints. The most common scenario of traumatic TFCC injury is falling on the pronated outstretched hand [18]. Avulsion of the articular disc (Figure 1E,F), rupture of the meniscus homologue, tears of the adjacent ligaments, or subluxation of the ECU tendon can be observed in affected patients.

There are two main mechanisms leading to TFCC degeneration. The first mechanism is repetitive pronation–supination as the axis of twisting passes through the articular disc [19]. The second mechanism is repetitive axial loading on the ulnar aspect of the wrist. The shape of the distal ulna plays a crucial role in TFCC injuries. Positive ulnar variance is associated with a higher risk of TFCC disorders [20]. Articular disc degeneration and chondromalacia of the ulna, lunate, and triquetrum cartilage are more prevalent in this group.

## 4. Physical Examination

The most common symptom of TFCC injuries is tenderness over the palmar side of the ECU tendon, i.e., “ulnar fovea sign” [21]. Further, patients may have pain at the ulnar aspect of the wrist during passive forearm rotation, handgrip strength decrease, limited range of motion during pronation-supination, or dorsal radioulnar joint instability [21]. The ulnocarpal stress test (TFCC grind test) is helpful for the diagnosis of TFCC injuries through induction of passive maximum ulnar deviation [22]. The screwdriver test applies an axial load on the ulnocarpal joint while rotating the forearm from full supination to pronation [23]. Ulnar wrist pain can be induced through both tests. In addition, DRUJ stability should be evaluated in TFCC injuries. The ballottement test can be performed by pushing the distal ulna to both dorsal and palmar directions after the radius is stabilized by the examiner. An increased displacement at the affected site compared with that at the contralateral wrist indicates DRUJ instability [24]. However, physical examination has limitations. All the aforementioned examinations cannot locate the exact lesion site. Therefore, further imaging studies are required in all cases with suspected TFCC disorders. 

## 5. Imaging (Other than US)

**Figure 2:**
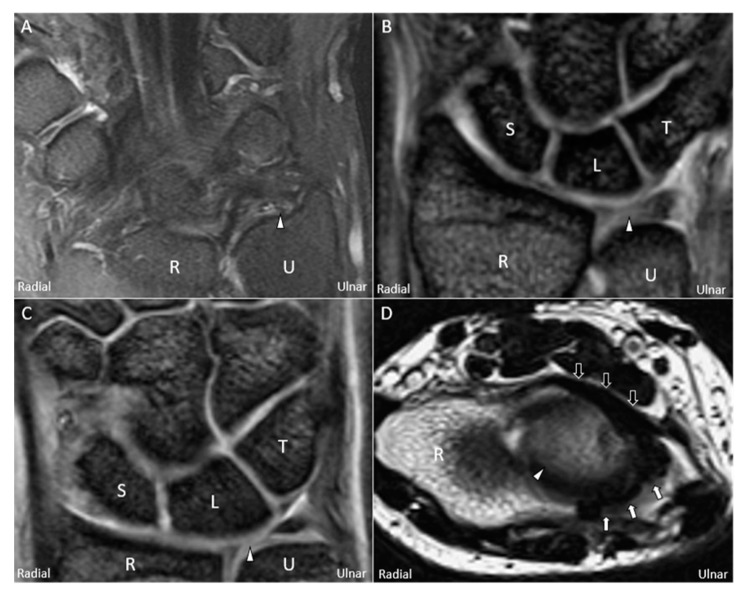
Magnetic resonance imaging (coronal T2-weighted view) showing (**A**) avulsion of the articular disc *(white arrowhead)* from the ulnar fovea, (**B**) central tear of the disc, and (**C**) radial avulsion of the articular disc. (**D**) Magnetic resonance imaging (axial T2-weighted view) showing a swollen palmar radioulnar ligament *(black arrow)* and a torn dorsal radioulnar ligament *(white arrow)* surrounding the articular disc. U: ulna; R: radius; L: lunate; T: triquetrum; S: scaphoid.

Plain radiography provides limited information on TFCC lesions, except for detection of positive ulnar variances, bony fractures, or DRUJ dislocations. Wrist arthrography is fairly accurate for diagnosing full-thickness TFCC injuries and is very useful in conjunction with MRI or MR arthrography, which is the imaging gold standard [25]. Notably, MRI is more applicable owing to its less invasiveness and has good accuracy in detecting articular disc lesions (Figure 2A–C), adjacent bony edema, adjacent ligament tears (Figure 2D), and cartilage degeneration [26]. The Palmer classification [1] is widely preferred for categorizing TFCC lesions using MRI, which is also helpful for guiding patient management [27].

## 6. US Imaging

**Figure 3:**
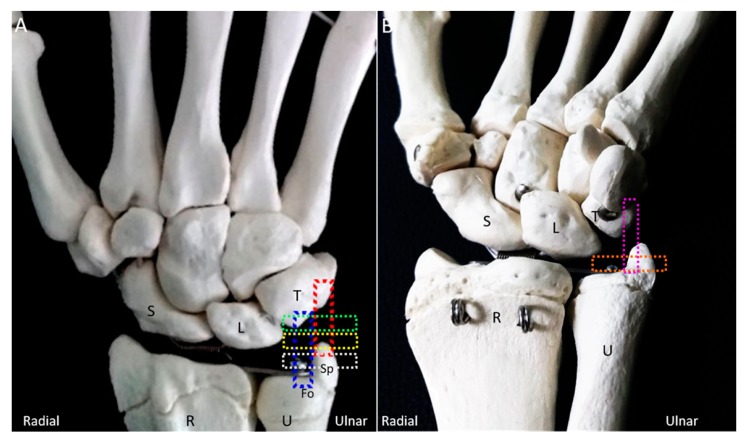
The colored dashed rectangles indicate the transducer positions on the (**A**) dorsal and (**B**) ventral aspects of the wrist for scanning the triangular fibrocartilage complex. U: ulna; R: radius; L: lunate; T: triquetrum; S: scaphoid; SP: styloid process; Fo: fovea.

High-resolution US is suitable for the evaluation of the TFCC (including its injuries) [28]; however, a single scanning view is insufficient for a substantial assessment. Therefore, familiarization with the TFCC anatomy under US imaging and following a systematic imaging protocol are invaluable in clinical practice. Herein, we proposed a comprehensive scanning method exploring different aspects of the TFCC (Figure 3A,B). All the US images were acquired using a 5-18-MHz linear transducer (HI VISION Ascendus, Hitachi, Japan).

### 6.1. Scanning Protocol

#### 6.1.1. Long-Axis View

**Figure 4:**
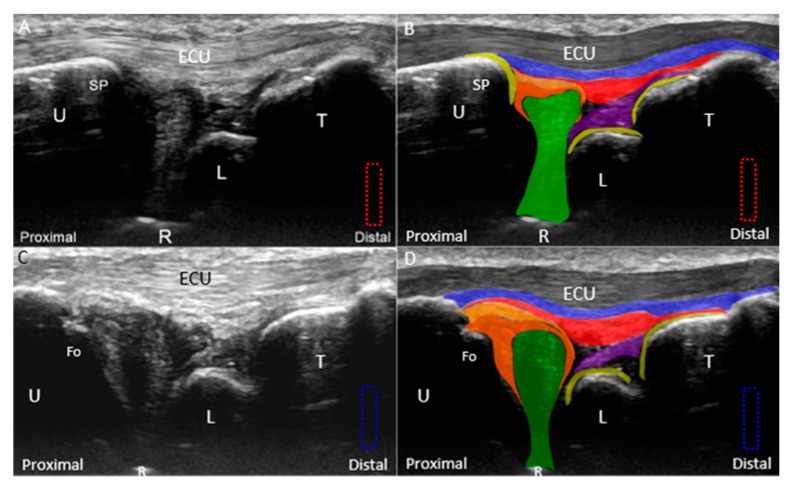
(**A**) Ultrasound (US) imaging of the triangular fibrocartilage complex (TFCC) using the long-axis approach; (**B**) superposed schematic drawing; (**C**) US imaging of the TFCC near the ulnar fovea using long-axis approach; and (**D**) superposed schematic drawing. The different color dashed rectangles are in accordance with the transducer positions in Figure 3. US: ultrasound; U: ulna; R: radius; L: lunate; T: triquetrum; SP: styloid process; Fo: fovea; ECU, extensor carpi ulnaris; TFCC: triangular fibrocartilage complex. Superficial limb of the radioulnar ligament *(light brown shade)*, deep limb of the radioulnar ligament *(dark brown shade)*, articular disc *(green shade)*, ECU tendon *(grey shade)*, ulnar collateral ligament *(blue shade)*, meniscus homologue *(red shade)*, lunotriquetral ligament *(purple shade)*, and cartilage *(yellow shade)*.

The wrist is placed on a pillow with the forearm in pronation and wrist in radial deviation. The transducer is positioned between the ulnar styloid process and the triquetrum, parallel to the hyperechoic ECU tendon. The ECU tendon, stabilized by the hypoechoic subsheath [29], is the most superficial structure of the TFCC and inserts on the fifth metacarpal base. The articular disc appears hypoechoic and tapers from its superficial portion to the deep attachment on the radius. The disc is encircled by the radioulnar ligament. However, the cranial aspect of the articular disc and part of the radioulnar ligament may be masked by the acoustic shadow of the ulnar styloid process. The ulnar collateral ligament, which links the distal ulna, hamate, and 5th metacarpal base, can be seen as a hyperechoic fibrillary structure interposed between the ECU tendon sheath and the radioulnar ligament. The lunate and triquetrum are covered by an anechoic thin layer of hyaline cartilage and are connected by a hyperechoic interosseous ligament. Dynamic US examination using radioulnar deviation can be performed to check the stability of the interosseous ligament (Video S1), superficial to which is the hammock-shaped meniscus homologue [30], appearing as a mixed hyperechoic and hypoechoic structure bridging the triquetrum and the articular disc (Figure 4A,B). The prestyloid recess, an extension of the radiocarpal joint (Figure 1A) [30], is a synovial space intercepting the disc and meniscus homologue. It is invisible in the absence of effusion.

By relocating the transducer radial to the ulnar styloid process, the ulnar fovea is seen as a small bony platform extending from the distal ulna. This approach is suitable for the evaluation of the ulnar attachment of the radioulnar ligament. The superficial limb of the radioulnar ligament can be seen inserting on the distal ulna. The deep limb of the radioulnar ligament encircles the articular disc and appears more hypoechoic than the superficial limb owing to the steeper insonation angle. It can be seen inserting on the ulnar fovea (Figure 4C,D).

**Figure 5:**
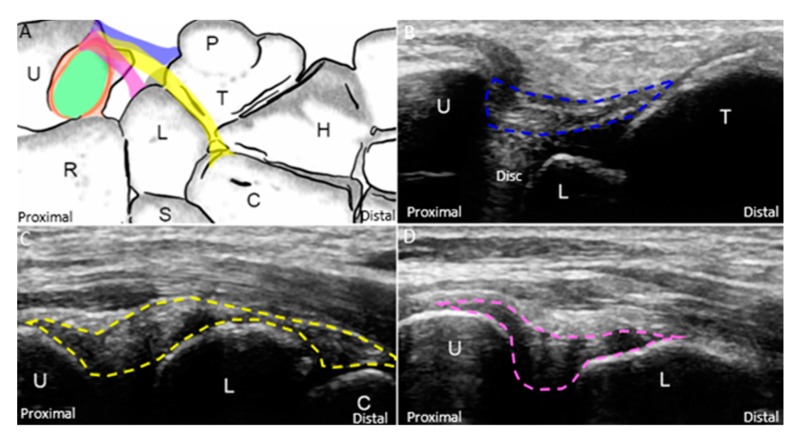
(**A**) The schematic drawing illustrates the ulnotriquetral *(blue band)*, ulnocapitate *(yellow band)*, and ulnolunate *(purple band)* ligaments. US imaging of the (**B**) ulnotriquetral *(blue dashed line)*, (**C**) ulnocapitate *(yellow dashed line)*, and (**D**) ulnolunate *(purple dashed line)* ligaments. US: ultrasound; U: ulna; R: radius; L: lunate; T: triquetrum; P: pisiform; H: hamate; C: capitate; S: scaphoid. Superficial limb of the radioulnar ligament *(light brown shade)*, deep limb of the radioulnar ligament *(dark brown shade)*, and articular disc *(green shade)*.

As for the ulnocarpal ligaments, the forearm should be supinated to allow the examination of the ventral wrist. The ulnotriquetral, ulnocapitate, and ulnolunate ligaments can be sequentially examined through pivoting the distal end of the transducer from the ulnar to the radial aspect (Figure 5A). The ulnotriquetral ligament can be visualized between the ulna and triquetrum and on top of the articular disc (Figure 5B). The ulnocapitate ligament passes the lunate and attaches to the capitate (Figure 5C). The ulnolunate ligament can be seen bridging the distal ulna and lunate (Figure 5D).

#### 6.1.2. Short-Axis View

**Figure 6:**
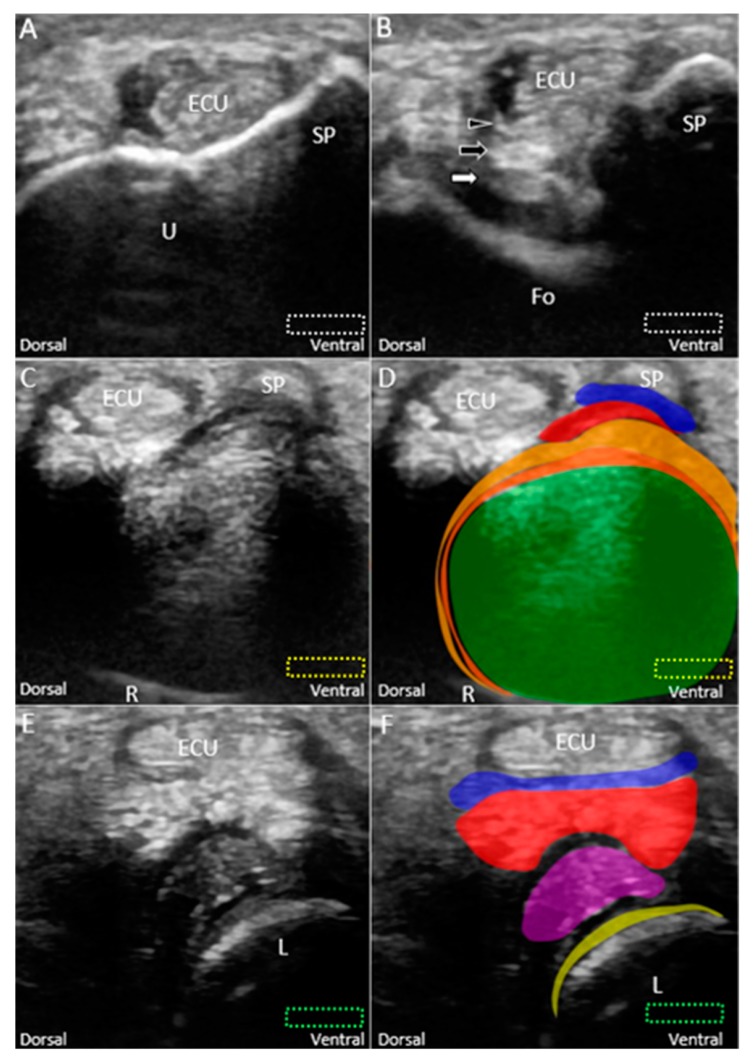
Imaging of the ulnar fovea using the short-axis approach. (**A**) The transducer is placed above the ulnar styloid process at first. It is then moved distally and tilted to the cranial aspect. (**B**) US imaging of the ulnar fovea shows the deep *(white arrow)* and superficial limbs *(black arrow)* of the radioulnar ligament and the ulnar collateral ligament *(black arrowhead)*. (**C**) US and (**D**) superposed US images of the articular disc above the sigmoid notch of the radius. (**E**) US and (**F**) superposed US images of the lunotriquetral ligament and meniscus homologue. The different color dashed rectangles are in accordance with the transducer positions in Figure 3. US: ultrasound; U: ulna; R: radius; L: lunate; SP: styloid process; Fo: fovea; ECU, extensor carpi ulnaris. Articular disc *(green shade)*, radioulnar ligament *(brown shade)*, ulnar collateral ligament *(blue shade)*, lunate cartilage *(yellow shade)*, ulnotriquetral ligament (*purple shade)*, and meniscus homologue *(red shade)*.

**Figure 7:**
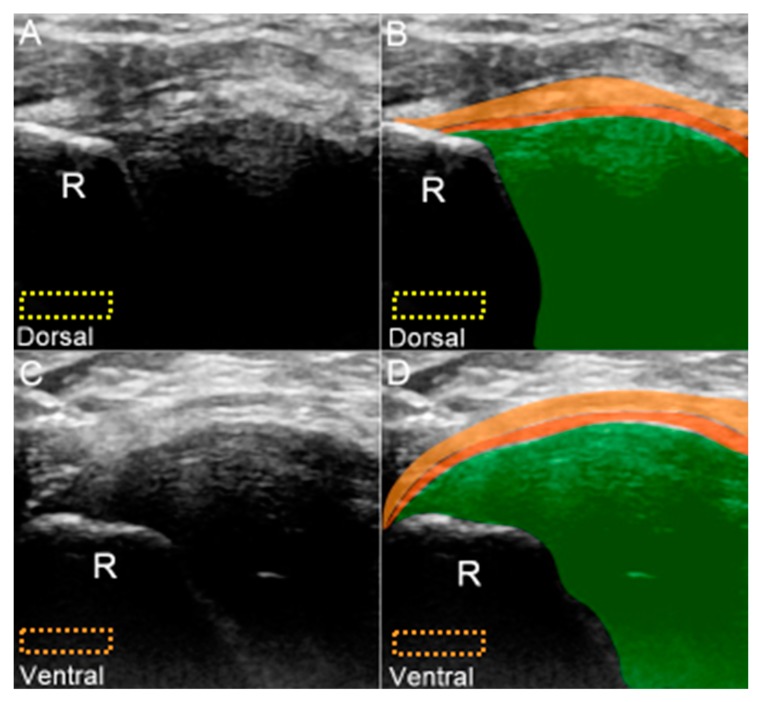
(**A**) US and (**B**) superposed US images of the dorsal radioulnar ligament. (**C**) US and (**D**) superposed US images of the ventral radioulnar ligament. The different color dashed rectangles are in accordance with the transducer positions in Figure 3. US: ultrasound; R: radius. Superficial limb of the radioulnar ligament *(light brown shade)*, deep limb of the radioulnar ligament *(dark brown shade)*, and articular disc *(green shade)*.

The transducer is placed at the ulnar wrist perpendicular to the ECU tendon (Figure 6A). Radial deviation of the wrist is helpful for visualization of the TFCC in cases with a narrow ulnocarpal space. To investigate the lesions near the ulnar fovea, the transducer should be placed in the horizontal plane distal to the ulnar styloid process and then tilted towards the distal ulnar articular circumference (Figure 6B). The bony platform of the ulnar fovea, the origin of the ulnar collateral ligament, and superficial and deep limbs of the radioulnar ligament can be viewed (Figure 6B). By gradually moving the transducer from proximal to distal, the circular-shaped articular disc above the sigmoid notch (medial aspect of the distal radius) is first seen (Figure 6C,D). When the transducer is moved more distally, the meniscus homologue and the underlying interosseous ligaments are visualized. The echogenicity of the interosseous ligament is lower than that of the meniscus homologue owing to the steep insonation angle on the oblique ligamentous fibers (Figure 6E,F). The longitudinal axis of the dorsal and palmar radioulnar ligaments can be seen encircling the articular disc from the dorsal or ventral aspect of the ulnar wrist (Figure 7A–D).

## 7. Pathologies—Classification

TFCC lesions include ruptures of the articular disc or meniscus homologue, disruption of the juxta-articular ligaments, and ECU tendinopathy. Normally, no Doppler signal is visualized inside the TFCC, although some tiny vessels may be found inside the adjacent ligaments [3]. In acute injuries, it is common to visualize an increase in vascularity inside the TFCC. In chronic cases, the individual components of the TFCC may not be well-demarcated owing to the presence of scars [31]. Thus, we also proposed a classification based on US findings for TFCC injuries modified from the Palmer classification [1] (Table 1). In our classification, the TFCC lesions can be divided into two major groups based on the underlying causes: Class 1 (trauma) and Class 2 (degeneration).

**Figure 8:**
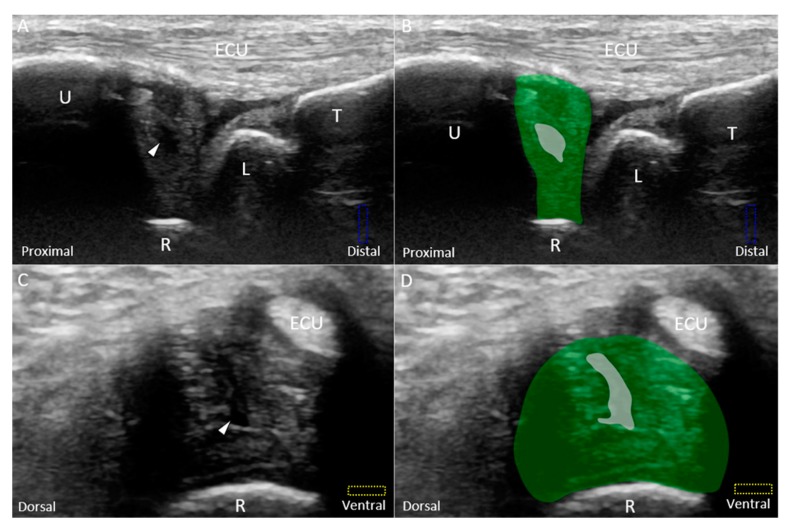
(**A**) US and (**B**) superposed US images using the long-axis approach show a central tear *(white arrowhead and grey shade)* of the articular disc *(green shade)*, compatible with Class 1A triangular fibrocartilage complex injury. (**C**) US and (**D**) superposed US images using the short-axis approach in the same patient. The different color dashed rectangles are in accordance with the transducer positions in Figure 3. US: ultrasound; U: ulna; R: radius; L: lunate; T: triquetrum; ECU, extensor carpi ulnaris.

**Figure 9:**
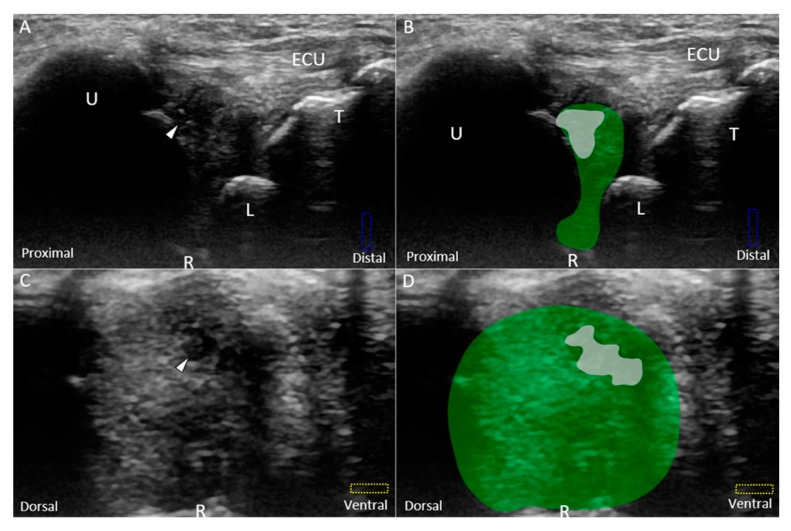
(**A**) US and (**B**) superposed US images using the long-axis approach show a tear *(white arrowhead and grey shade)* of the disc *(green shade)* near the ulnar attachment, indicating Class 1B triangular fibrocartilage complex injury. (**C**) US and (**D**) superposed US images using the short-axis approach show a hypoechoic lesion in the disc as well. The different color dashed rectangles are in accordance with the transducer positions in Figure 3. US: ultrasound; U: ulna; R: radius; L: lunate; T: triquetrum; SP: styloid process; ECU, extensor carpi ulnaris.

**Figure 10:**
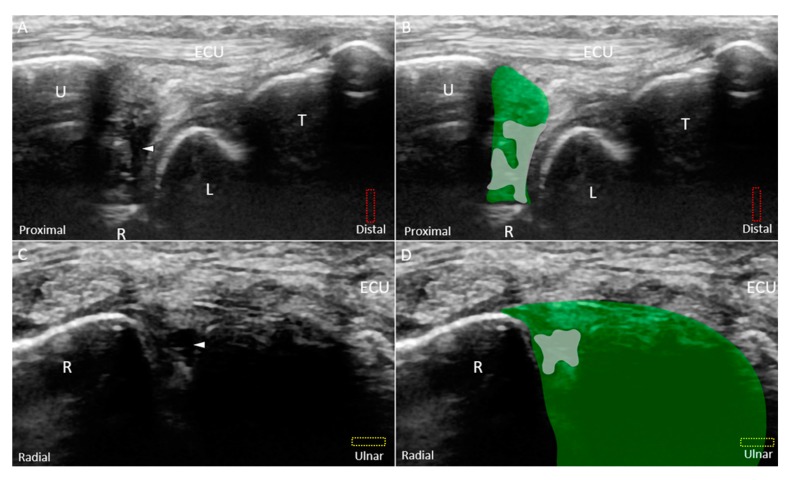
(**A**) US and (**B**) superposed US images using the long-axis approach show a tear *(white arrowhead and grey shade)* of the disc *(green)* emerging from the radius, indicating Class 1C triangular fibrocartilage complex injury. (**C**) US and (**D**) superposed US images using the short-axis approach in the same patient. The different color dashed rectangles are in accordance with the transducer positions in Figure 3. US: ultrasound; U: ulna; R: radius; L: lunate; T: triquetrum; ECU, extensor carpi ulnaris.

**Figure 11:**
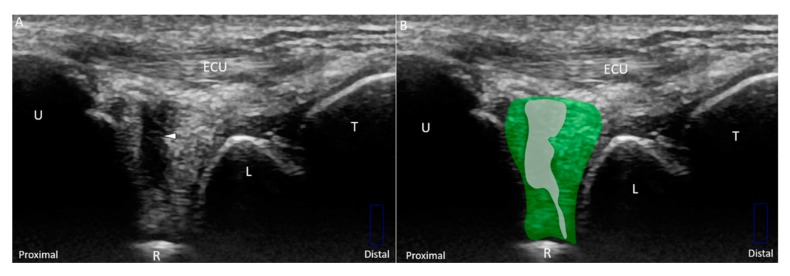
(**A**) US and (**B**) superposed US images show a longitudinal tear *(white arrowhead and grey shade)* extending through the entire diameter of the disc *(green shade)*, indicating Class 1D triangular fibrocartilage complex injury. The different color dashed rectangles are in accordance with the transducer positions in Figure 3. US: ultrasound; U: ulna; R: radius; L: lunate; T: triquetrum; ECU, extensor carpi ulnaris.

Class 1A–D injuries are related to articular disc ruptures. The articular disc, interposed between the lunate and ulna, has a concave lens-like shape (thicker peripherally and thinner centrally) [32]. Class 1A injuries refer to central tears in the articular disc. US imaging reveals a hypoechoic cleft at the disc’s middle portion without affecting its entire diameter (Figure 8A–D). Class 1B injuries, which include articular disc tears at the insertion of the distal ulna, are the most prevalent type of Class 1 injuries (Figure 9A–D) [33] and are associated with ulnar styloid fractures [29]. If the lesions involve the ulnar fovea, DRUJ instability may coexist [34]. Class 1C injuries indicate a tear of the articular disc near its radial attachment. US imaging shows a hypoechoic slit emerging from the floor of the TFCC (Figure 10A–D). Class 1D injuries indicate a tear extending through the entire depth of the articular disc (Figure 11A,B).

**Figure 12:**
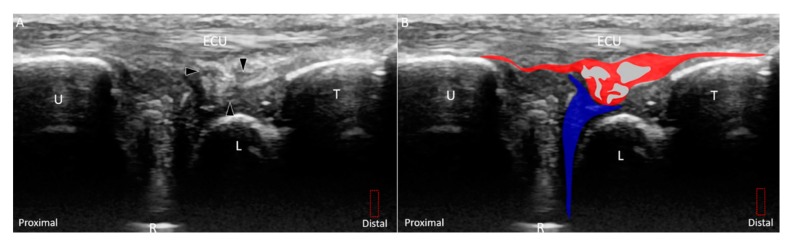
(**A**) US and (**B**) superposed US images using the long-axis approach show tears *(black arrowhead and grey shade)* of the meniscus homologue *(red shade)* and prestyloid recess effusion *(blue shade)*, indicating Class 1E triangular fibrocartilage complex injury. The different color dashed rectangles are in accordance with the transducer positions in Figure 3. US: ultrasound; U: ulna; R: radius; L: lunate; T: triquetrum; ECU, extensor carpi ulnaris.

**Figure 13:**
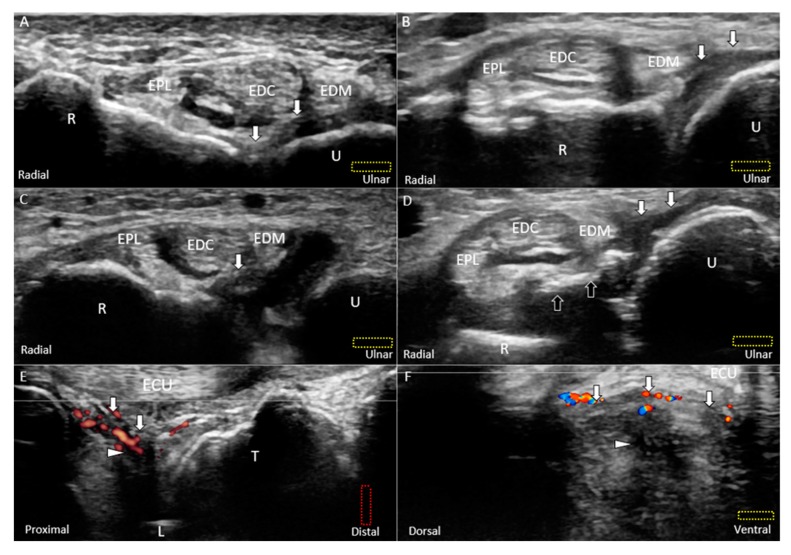
US images (short-axis view) of the (**A**) normal, (**B**) partially torn, (**C**) completely torn, and (**D**) avulsed dorsal radioulnar ligaments *(white arrow)*. Doppler US imaging of the inflamed dorsal radioulnar ligaments and central tear of the articular disc *(white arrowhead)* using the (**E**) long-axis and (**F**) short-axis views. All lesions are categorized under Class 1E injuries. The different color dashed rectangles are in accordance with the transducer positions in Figure 3. US: ultrasound; black arrow: avulsed bony fragment from the radius; U: ulna; R: radius; L: lunate; T: triquetrum; EPL: extensor pollicis longus; EDC: extensor digitorum communis; EDM: extensor digiti minimi; ECU: extensor carpi ulnaris.

**Figure 14:**
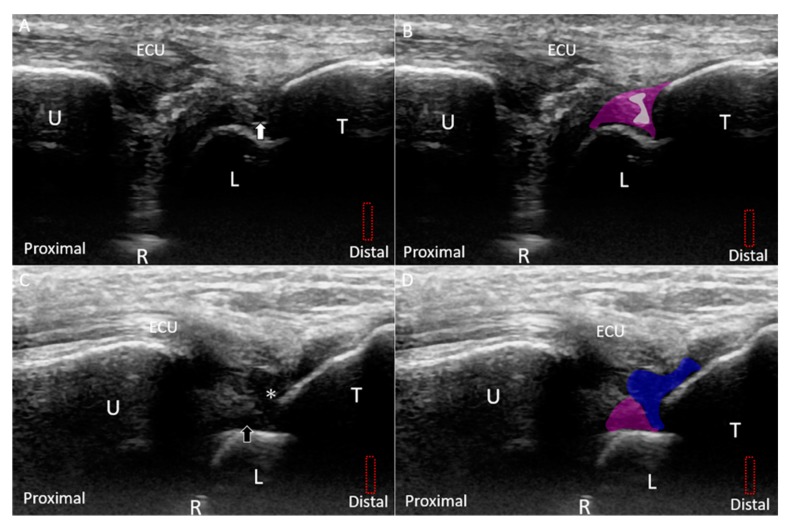
(**A**) US and (**B**) superposed US images show a small tear *(white arrow and grey shade)* in the lunotriquetral ligament *(purple shade)* under the long-axis approach. (**C**) US and (**D**) superposed US images of the retracted stump *(white asterisk and blue shade)* of the torn lunotriquetral ligament and fluid accumulation *(black arrow and purple shade)*. The different color dashed rectangles are in accordance with the transducer positions in Figure 3. US: ultrasound; U: ulna; R: radius; L: lunate; T: triquetrum; ECU, extensor carpi ulnaris.

**Figure 15:**
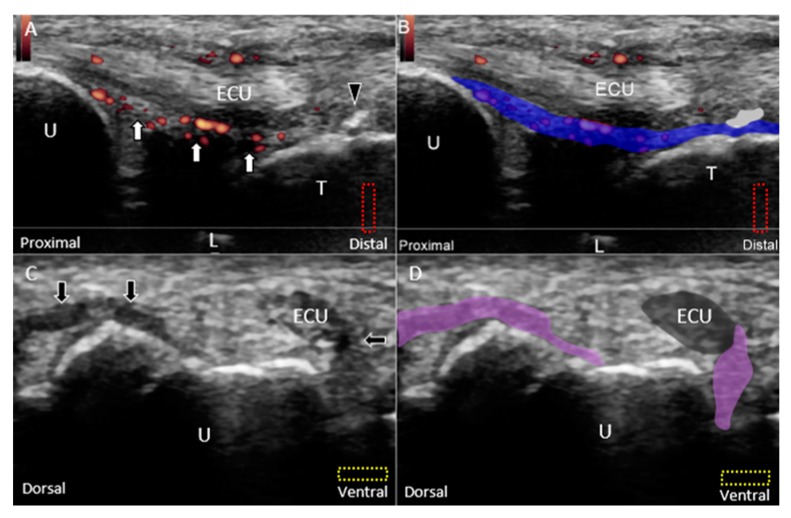
(**A**) Doppler US and (**B**) superposed images show the hypervascular and swollen ulnar collateral ligament *(white arrow and blue shade)* and ECU tendon as well as an avulsed fragment of the triquetrum *(black arrowhead and grey shade)* under the long-axis approach. (**C**) US and (**D**) superposed US images show rupture of the ECU tendon subsheath *(black arrow and purple shade)* with subluxation of the extensor carpi ulnaris (ECU) tendon *(dark grey shade)* using the short-axis approach. The different color dashed rectangles are in accordance with the transducer positions in Figure 3. US: ultrasound; U: ulna; L: lunate; T: triquetrum; ECU: extensor carpi ulnaris.

Class 1E injuries, a newly added category to the original Palmer classification based on MRI findings, refer to the involvement of the TFCC other than the articular disc. The subgroup includes injury of the meniscus homologue, which may have a heterogeneous echotexture owing to anisotropy from its peripheral fibers arising from the surrounding ligaments [35]. However, when the meniscus homologue is truly damaged, its echogenicity becomes more inhomogeneous, especially at its central portion (Figure 12A,B). Injuries of the radioulnar (Figure 13A–F), lunotriquetral (Figure 14A–D), and ulnar collateral (Figure 15A,B) ligaments are also common in this subgroup. Under US imaging, the affected ligaments may lose their fibrillary pattern, become wavy, and even retract with formation of adjacent hematomas. In cases with distal radioulnar joint instability, the dorsal and palmar radioulnar ligaments should be examined using dynamic US. In some cases, the ECU tendon subsheath is affected, leading to tendon subluxation (Figure 15C,D).

**Figure 16:**
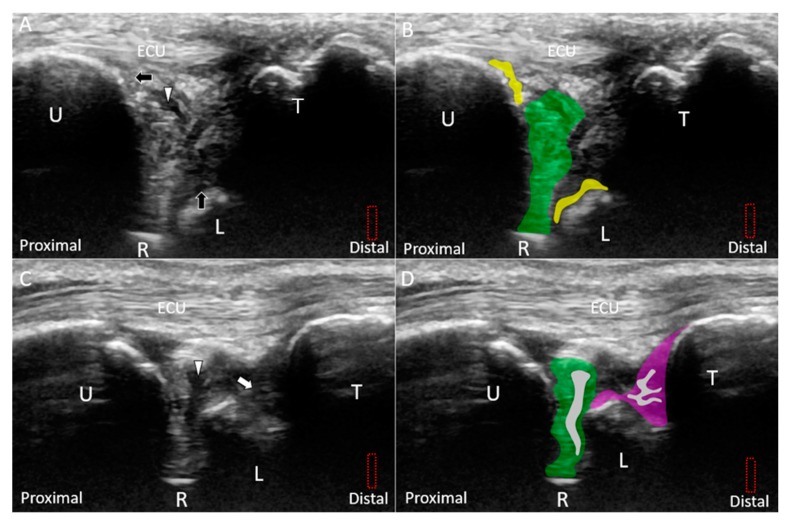
(**A**) US and (**B**) superposed US images show a peripheral tear *(white arrowhead)* of an irregularly demarcated articular disc *(green shade)* and mild cartilage erosion of the lunate and ulna *(black arrow and yellow shade)* under the long-axis approach, indicating Class 2B TFCC injury. (**C**) US and (**D**) superposed US images show a large central tear *(white arrowhead and grey shade)* of a thin articular disc *(green shade)* and rupture *(white arrow and grey shade)* of the lunotriquetral ligament *(purple shade)*, indicating Class 2D TFCC injury. The different color dashed rectangles are in accordance with the transducer positions in Figure 3. US: ultrasound; U: ulna; R: radius; L: lunate; T: triquetrum; ECU: extensor carpi ulnaris; TFCC: triangular fibrocartilage complex.

Class 2 injuries indicate degeneration of the TFCC with or without precedent trauma [1]. The severity is incremental from Class 2A to 2E [36]. Class 2A injuries indicate wear of the articular disc. US imaging may reveal that the articular disc at the affected site becomes thinner and has a more irregular border than that at the contralateral site. Class 2B injuries indicate articular disc wear with adjacent chondromalacia demonstrated by thinning of the articular disc and juxta-articular cartilage and irregular cortex of the adjacent carpal bones (Figure 16A,B). Class 2C injuries are similar to Class 2B injuries but may progress to disc rupture. Class 2D injuries refer to additional lunotriquetral ligament ruptures (Figure 16C,D). The most severe type is Class 2E injuries, suggesting arthritis and synovitis of the ulnocarpal and radioulnar joints with a large tear of the articular disc [29].

## 8. Treatment

### 8.1. General Principles

The conservative treatments for TFCC injuries include prescription of analgesics, rest, kinesiotaping, and immobilization. If the symptoms cannot be relieved, an injection can be considered. For acute injuries with hypervascularity over the TFCC under US imaging, a small amount of corticosteroid (e.g., 10 mg *triamcinolone*) can be injected near the lesion to decrease inflammatory pain. For severe cases, surgical repair with or without ulnar-shortening osteotomy should be taken into consideration. Rupture at the peripheral part of the TFCC has a better surgical outcome than that at its central part or near its radial insertion [34]. However, for injuries near the fovea, surgical repair is difficult and often has an unsatisfactory outcome [37]. After the surgery, the wrist should be immobilized for 4 to 6 weeks followed by rehabilitation.

### 8.2. US-Guided Injection

The TFCC is predominantly innervated by the dorsal ulnar cutaneous nerve [38]. Its blood supply arises from the ulnar artery and branches of the anterior or posterior interosseous artery [39,40]. The abovementioned structures should be clearly identified and prevented from acquiring any accidental injury during the injection.

**Figure 17:**
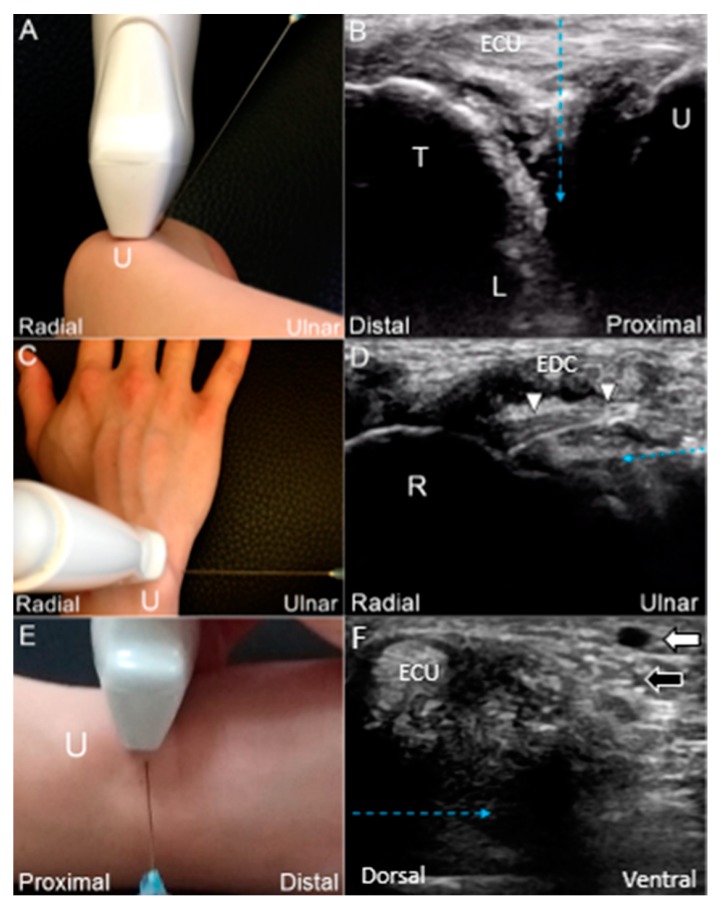
(**A**) US-guided injection using the *out-of-plane* approach. (**B**) The needle *(blue dashed arrow)* is advanced toward the radial aspect, deeper to the ECU tendon. (**C**) US-guided injection using the *in-plane* approach from the ulnar wrist. (**D**) The needle is advanced toward the radial aspect, deeper to the dorsal radioulnar ligament *(white arrowhead)*. (**E**) US-guided injection using the *in-plane* approach from the dorsal wrist. (**F**) The needle is advanced toward the ventral aspect, deeper to the ECU tendon. US: ultrasound; U: ulna; R: radius; L: lunate; T: triquetrum; white arrow: artery; black arrow: dorsal ulnar cutaneous nerve; ECU: extensor carpi ulnaris; EDC: extensor digitorum communis.

For US-guided injection of the TFCC using the *out-of-plane* approach (Figure 17A,B; Video S2), the wrist is placed on a pillow with the forearm fully pronated and the wrist radially deviated. The ulnar styloid process is faced upward. The linear transducer is placed parallel to the ECU tendon between the ulnar styloid process and triquetrum. A 25-gauge needle is advanced toward the radial aspect deeper to the ECU tendon until the needle tip reaches the target.

If the patient cannot fully pronate the forearm or the ulnocarpal space is narrow, US-guided injection using the *in-plane* approach is the best option. The wrist is placed on a pillow with the forearm pronated and wrist radially deviated. The linear transducer is placed over the dorsal wrist in the horizontal plane. A 25-gauge needle is advanced toward the radial aspect deeper to the ECU tendon for reaching the TFCC (Figure 17C,D; Video S3). For injecting to the radial attachment of the articular disc, another *in-plane* technique would be useful. The linear transducer is placed at the ulnar wrist perpendicular to the ECU tendon. A 25-gauge needle is advanced in a ventral direction deeper to the ECU tendon to target the TFCC (Figure 17E,F).

## 9. Conclusions

The TFCC is a complex structure; following a systematic approach, US can well depict different components of the TFCC and elaborate the lesions of the articular disc, meniscus homologue, and juxta-articular ligaments. In cases of TFCC disorders, US can also help in precise delivery of injectates to the injured parts and thus reduce collateral damage to adjacent nerves and vessels.

## Figures and Tables

**Table 1:** Ultrasound classification for triangular fibrocartilage complex lesions.

Class 1 (Traumatic)
(A). Central tear of the articular disc
(B). Ulnar attachment tear of the articular disc
(C). Radial attachment tear of the articular disc
(D). Horizontal tear of the articular disc
(E). Injury of the triangular fibrocartilage component other than the articular disc, i.e., meniscus homologue; radioulnar, lunotriquetral, and ulnar collateral ligaments; or extensor carpi ulnaris tendon subsheath
**Class 2 (Degenerative)**
(A). Articular disc wear
(B). Class 2A plus chondromalacia of the lunate or the ulna
(C). Class 2B plus articular disc rupture
(D). Class 2C plus lunotriquetral ligament rupture and cartilage abnormalities
(E). Class 2D plus ulnocarpal/radioulnar arthritis

## Supplementary Materials

The following are available online at https://www.mdpi.com/2077-0383/8/10/1540/s1, Video S1: dynamic ultrasound examination for the triangular fibrocartilage complex; Video S2: ultrasound guided *out-of-plane* injection technique for the triangular fibrocartilage complex; Video S3: ultrasound guided *in-plane* injection technique for the triangular fibrocartilage complex.
